# Editor's Letter-Box

**Published:** 1900-06-30

**Authors:** 


### EDITOR'S LETTER-BOX.

[Our Correspondents are reminded that prolixity is a great bar to publication, and that brevity of style and conciseness of statement greatly facilitate early insertion.]

Our correspondent from Dublin on "Young Criminals" is reminded that we cannot publish any letter unless we have, for our private information, the full address of the writer.

